# Changes in heart failure healthcare in general practice during the COVID-19 pandemic: a survey

**DOI:** 10.3399/BJGPO.2024.0138

**Published:** 2025-09-10

**Authors:** Raphael Rothenberger, Thomas Blakeman, Carolyn A Chew-Graham, Faye Forsyth, Muhammad Hossain, Emma Sowden, Christi Deaton

**Affiliations:** 1 Department of Cardiology, University Hospital Bern, Bern, Switzerland; 2 Harvard T.H. Chan School of Public Health, Boston, United States; 3 Centre for Primary Care and Health Services Research, School of Health Sciences, The University of Manchester Division of Population Health Health Services Research and Primary Care, Manchester, United Kingdom; 4 Faculty of Medicine and Health Sciences, Keele University, Newcastle-under-Lyme, United Kingdom; 5 Department of Public Health and Primary Care, KU Leuven, Leuven, Belgium; 6 Department of Primary Care and Public Health, University of Cambridge School of Clinical Medicine, Cambridge, United Kingdom; 7 School of Health Sciences, Birmingham City University, Birmingham, United Kingdom; 8 Division of Nursing, Midwifery and Social Work, University of Manchester, Manchester, United Kingdom

**Keywords:** general practice, heart failure, remote consultations

## Abstract

**Background:**

The COVID-19 pandemic accelerated the adoption of remote healthcare for the management of long-term conditions, including heart failure (HF). However, little is known about the experiences of this transition, knowledge developed during service transformation, and/or the preferences of clinicians and patients with HF going forward.

**Aim:**

This study aimed to determine the perspectives and consensus of healthcare providers, patients, and carers in the UK regarding the transition to remote healthcare for HF during the pandemic.

**Design & setting:**

A survey was conducted among individuals with HF and healthcare providers.

**Method:**

An evaluation of stakeholders’ views and a consensus-development exercise regarding the use of telehealth and home monitoring devices were conducted using a modified Delphi process on an online platform. This study primarily included patients with HF with preserved ejection fraction (HFpEF).

**Results:**

Findings revealed a significant reliance on telehealth, which reportedly enhanced accessibility for patients. Integration of home monitoring devices and systems was deemed crucial for continuity of care. Although there was some consensus concerning the usefulness of remote consultations and communication, findings also highlighted their limitations and disparities in digital access and literacy.

**Conclusion:**

Remote healthcare presents opportunities and risks, emphasising the need for equitable access to telehealth technologies and thoughtful integration into healthcare systems. Balancing remote and in-person care, along with targeted training for healthcare providers, is essential for effective management and support of people with long-term conditions.

## How this fits in

With the rise of the use of remote healthcare, clinicians should be aware of its challenges and how to balance remote and in-person care to manage long-term conditions effectively and equitably. This study sheds light on healthcare providers, patients, and carers’ perspectives of remote care in heart failure during the COVID-19 pandemic in the UK.

## Introduction

The COVID-19 pandemic and the resulting restrictions imposed on the population resulted in major changes to healthcare delivery and healthcare experiences.^1,2^ Elective procedures and outpatient appointments were cancelled, and many specialist services closed or operated at a reduced capacity because of the redeployment of medical, nursing, and therapy staff to care for patients with COVID-19^3,4^. Before the pandemic, efforts to integrate alternative consultation methods beyond face-to-face interactions within UK general practices constituted a nuanced landscape characterised by incremental adoption and variable perceptions of patient suitability, stressing the need for engagement of all staff members.^5^ As a result of the pandemic, general practices and specialist clinics shifted from in-person to remote telephone and video consultations,^6^ allowing responses to practice-related queries to be provided directly by clinicians without necessitating face-to-face evaluation in the clinical setting, which carried a possible risk of infection^7^. Further, people were told to ‘protect the NHS’, and many reacted by not using services. People with heart failure (HF) began or increased home monitoring using blood pressure cuffs, oxygen saturation monitors, and other technologies.^6^

Restrictions resulting from the COVID-19 pandemic led to missed opportunities for early diagnosis,^8^ a backlog of elective procedures,^9^ and deterioration in patients with long-term conditions because of a lack of usual monitoring. The British Heart Foundation (BHF) reported over 5000 excess deaths as a result of HF in England between March 2021 and March 2022;^10^ similar trends were seen in other countries ^11^. Responses from the BHF patient survey indicated that 43% of patients who required care for their heart condition put off seeking help primarily to avoid putting pressure on the NHS.^10^ However, the challenges of reduced access and preventing infection also led to innovation and changes^6^ that could form part of the solution to the continuing crisis in access. The complex nature of HF, coupled with multimorbidity and social factors, presents a distinct challenge to implementing remote care models. Addressing this needs research that extends beyond the scope of traditional clinical trials to better reflect real-world settings.^12^ This study aimed to determine, through established consensus methods, whether people with HF and clinicians could reach an agreement on which innovations might be maintained.

## Method

### Study design and ethics

This study used a modified online Delphi process,^13^ which is a structured communication technique applying iterative survey rounds to gather consensus, often used to develop best practice guidance.^14^ Two to three survey rounds were planned, depending on the degree of agreement. Approval for the recruitment of healthcare providers (HCPs) and patients through social media was received from the University of Cambridge Human Biology Research Ethics Committee. Approval for patients in the Optimise HFpEF study to be invited to participate was approved via North East–York Research Ethics Committee (REC), and London–Surrey REC approved amendments to two workpackages (REC references: 17/NE/0199 and 17/LO/2136). Written informed consent or e-consent were required for participation via the online platform, Thiscovery. The survey was conducted between December 2020 and March 2021.

## Survey development

The survey items were developed by investigators using information and insights from a qualitative study of primary care and specialist services and patients with HF, primarily with preserved ejection fraction (HFpEF).^6^ The survey was developed in collaboration with The Healthcare Improvement Studies Institute (THIS Institute) using their platform Thiscovery. Questions were presented in a variety of formats (open-ended, ranking). For example, one question for providers was: ‘During a pandemic, evidence indicates that there are delays in diagnosis of and treatment for heart failure and other long-term conditions. Please rate the importance of each item below in mitigating delay.’

Only minimal demographic information was collected. Most questions asked participants to rate specific items from 1 (not important at all) to 9 (extremely important), and some used word anchors such as ‘not confident at all’ to ‘extremely confident’ or considered the trustworthiness of various sources of information.

### Consensus-building process

Summary statistics were calculated for each item rated. For questions rated using a numerical scale from 1 to 9, we used a median score of 7 or greater (indicating mostly positive opinions) or 3 or less (indicating mostly negative opinions) to indicate consensus. A median of 7 or more meant that more than 50% of the participants scored the question as 7, 8, or 9. Conversely, a median of 3 indicated that more than 50% of participants gave low ratings of 3, 2, or 1. Disagreement was defined as more than one-third of respondents rating the statement at the opposite end of the scale to their peers.^15^ We undertook a consensus-building process to gain an understanding of the assessment of different aspects by HCPs and patients, with questions that did not reach the threshold for consensus in the first round being included in the second round, which was sent to all participants. Each participant was provided with their own score in the first round and the anonymised scores of the entire group. A few questions pertained only to patients or carers respectively, and a few only to HCPs. Otherwise, the questions were the same, with the wording changed to reflect whether they addressed patient or provider. Upon entering the Thiscovery platform, participants were directed to the appropriate version of the survey. HCPs were asked to indicate their profession and discipline. Personal data on Thiscovery was protected and not shared with investigators. In keeping with our ethical approval, people in the Optimise HFpEF study completed the survey rounds using the survey platform, Qualtrics, by the Optimise HFpEF investigators. Options for use of paper questionnaires and of support in answering the questions were also offered to patients invited by the Optimise HFpEF investigators.

### Participant recruitment

HCPs were recruited through professional networks, snowball contacts, and social media. Three clinical commissioning groups (CCGs) in the three regions where investigators were based (Cambridgeshire, Greater Manchester and West Midlands) distributed the survey link to all primary care practices in their areas (approximately 100 practices). CCGs were NHS bodies responsible for commissioning healthcare services, dissolved in 2022, to be replaced by primary care networks and integrated care boards. Members of the research team also invited specific primary care practices and GP groups known to them to participate. Primary care practices are the first point of contact with the NHS. GP groups are organised groups of GPs who work within a primary care practice. Approximately 40 GPs were invited and asked to cascade the survey link to colleagues. GP trainees at two universities were invited by a GP trainee colleague. The invitation and survey link were also disseminated at the Society for Academic Primary Care East conference.

Specialist HCPs were invited and asked to cascade the survey using the investigators’ professional contacts and through cardiology and geriatric teams at several hospitals. Specialist providers who had participated in Optimise HFpEF were also invited to participate. Social media was used to advertise the survey widely. The survey invitation and link were posted on three university healthcare professional websites. An invitation and survey link were sent to a relevant patient charity, and 46 patients who had participated in Optimise HFpEF were also invited to take part. Healthcare providers were asked to provide survey invitations, information, and links to appropriate patients.

## Results

Forty-three participants were included in the first round, comprising 22 HCPs, 20 patients, and one carer. Demographic characteristics are presented in Table 1. A few participants who were uncomfortable with using online questionnaires completed the survey on paper, which was subsequently transferred online by Optimise HFpEF investigators, and one person completed the survey with telephone support. In round 2, 12 HCPs, 11 patients, and one carer repeated the exercise on questions that had failed to gain consensus in the first round.

**Table 1.** Participant characteristics

	HCPs (*N*=22)	Patients and carers (*N*=21)
Profession	GP=15 GP trainee=1 Cardiologist=3 Nurse=3 (one each of PN, HFSN, and academic)	
Age	21–30 years=2 31–40 years=10 41–50 years=7 51–60 years=3	31–40 years=2 61–70 years=7 71–80 years=5 81–90 years=2 Not disclosed=5
Gender	Female=15 Male=7	Female=7 Male=10 Not disclosed=4
Geographical area	East of England=8 North West=5 West Midlands=3 Yorkshire & Humber=1 SouthWest=1 Outside of England=3	East of England=11 NorthWest=6 East Midlands=1 Not disclosed=2
Living situation		Live alone=3 Live with spouse only=12 Live with multiple family members=4 Live with friends=1 Not disclosed=1
Number of long-term conditions		One to two=5 Three to five=12 Six to eight=3 Not disclosed=1

GP = general practitioner, HCP = healthcare provider, HFSN = heart failure specialist nurse, PN = practice nurse.

### Provider perspectives

**Figure 1.**
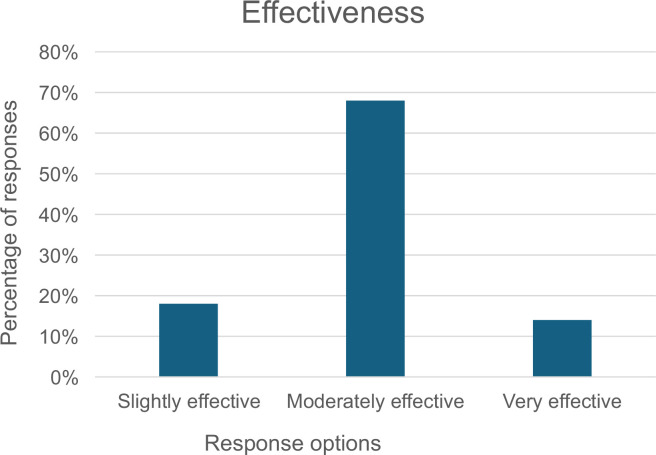
Provider assessment of the effectiveness of remote consultations

**Figure 2.**
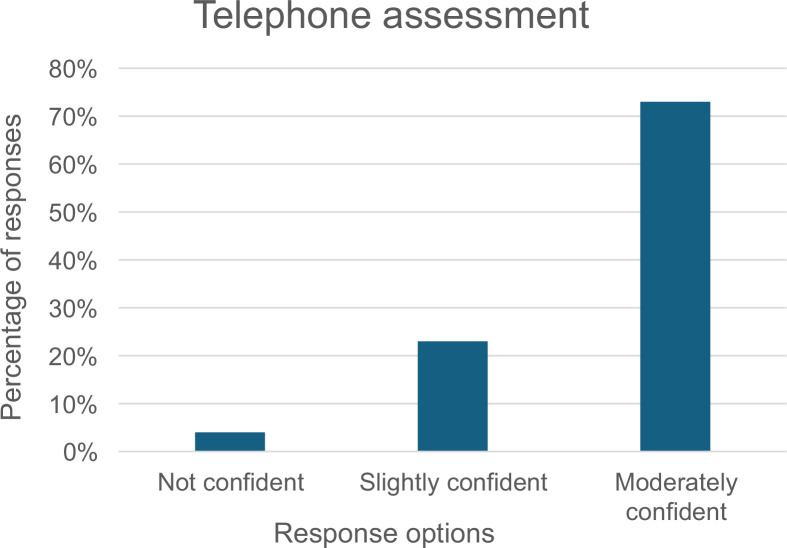
Provider confidence in using telephone assessment for patient care

Providers were asked about the effectiveness of remote consultations, ie, telephone or video platforms, for patients with long-term conditions and their confidence in assessing patients with HF over the telephone. The majority (68%) thought remote consultations were moderately effective and noted moderate confidence in telephone assessment (Figures 1 and 2).

HCPs were then asked to rate items related to the challenges of remote consultation and home monitoring devices and systems for patients (Table S1, supplementary material). The importance of monitoring devices and systems for patients varied, with pulse oximetry considered important (median 7.5, interquartile range (IQR) 6.0–9.0) but disagreement for ECG devices (median 5.0, IQR 2.0–7.0) and automated data transfer (median 6.0, IQR 4.0–8.0), potentially reflecting differences in practice among participants and their organisations. In times of a high volume of COVID-19 cases, specialist services for HF may be curtailed, so providers were asked about strategies when this occurred. Secure information transfer, items that reflected clear information (eg, criteria for referral and plan for care if patient discharged) were highly agreed, but there was no consensus on whether remote consultation should involve primary and secondary care providers together on either round.

### Patient perspectives

**Figure 3.**
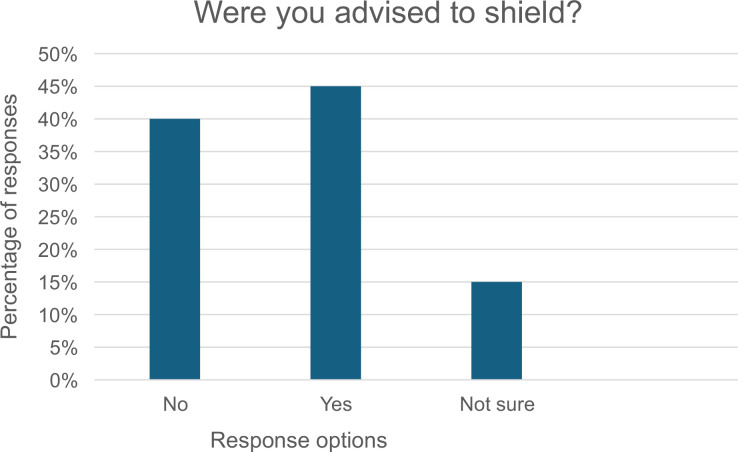
Responses to heart failure awareness and COVID-19 shielding advice among people with heart failure

People with HF were asked if they were advised to shield or told that they were clinically vulnerable to COVID-19. During COVID-19, patients considered to be vulnerable, ie, at the highest risk of severe illness if they contracted COVID-19, were advised to shield to protect themselves from the virus by staying home and avoiding face-to-face contact with others beyond a very small number of family members or a close friend. As can be seen in Figure 3, the response was almost evenly divided among those who were advised to shield (45%) and those not advised to do so (40%), with another 15% unsure. They were asked to rate the importance of factors influencing their decisions to shield if they did so and the trustworthiness of various sources of information about COVID-19 and risk (Table 2). Patients and carers were asked about their confidence in having healthcare needs met, revealing low trust in the system’s ability (median 3.0, IQR 2.2–4.0).

**Table 2.** Questions that were only asked of people with heart failure

Questions for patients	Patient median score (IQR) round 1	Patient median score (IQR) round 2 if applicable
**Please rate the importance of each item below in influencing your decision to shield**		
GPs recommendation	8.0 (1.0–9.0)	Consensus reached
Specialist healthcare provider recommendation	6.0 (1.0–9.0)	7.0 (2.0–7.50)
Decided to do without a recommendation from NHS or HCP	6.0 (1.5–9.0)	9.0 (7.0–9.0)
Knowledge of age and illness	8.5 (7.0–9.0)	Consensus reached
Letter received from NHS	6.0 (2.0–9.0)	5.0 (2.5–9.0)
Guidance received from NHS on shielding criteria	7.0 (3.0–9.0)	Consensus reached
Living with someone who needed to shield	5.0 (1.0–9.0)	8.0 (5.2–9.0)
**Information about COVID-19 and its risks has come from multiple sources. Please rate the trustworthiness of information from these sources from 1 not trustworthy at all to 9 extremely trustworthy**		
General practice	8.0 (6.0–9.0)	Consensus reached
Specialist clinic at hospital	9.0 (7.0–9.0)	Consensus reached
HFSN	8.0 (1.0–9.0)	Consensus reached
NHS England	7.0 (6.0–8.0)	Consensus reached
Internet sources	5.0 (2.0–7.0)	5.0 (3.0–5.0)
District council	4.0 (1.0–6.75)	5.0 (4.0–8.0)
Healthcare journals	3.0 (1.0–6.0)	Consensus reached
Newspaper	4.0 (1.0–6.0)	4.0 (1.5–5.7)
Television news	6.0 (2.0–7.0)	5.5 (3.2–7.0)
Social media	1.0 (1.0–3.75)	Consensus reached

HCP = healthcare provider, HFSN = heart failure specialist nurse

### Perspectives of people with HF and healthcare providers

Questions given to patients and HCPs about supported self-management, means of consultation and communication in times of limited healthcare access, and changes in healthcare that should be sustained showed little disagreement (Table 3).

**Table 3.** Questions asked of both people with HF and healthcare providers

Question	Score round 1 Median (IQR)	Score round 2 if applicable
**Supported self-management (eg, education and guidance from clinicians) has received increasing emphasis given reduced face-to-face consultations. Please rate the importance of each item below**		
Supplying free devices such as weighing scales and blood pressure monitors	8.0 (5.0–9.0)	Consensus reached
Linking devices to a telemonitoring system	7.0 (3.5–9.0)	Consensus reached
Patient willingness and ability to record and share information	8.0 (7.0–9.0)	Consensus reached
Access to providers trained to support home monitoring and self-management	8.0 (5.0–9.0)	Consensus reached
Supporting patients to know and understand their diagnosis of heart failure, including type (reduced or preserved ejection fraction)	8.0 (7.0–9.0)	Consensus reached
Safe space in hospital or practice for assessment of patients when self-monitoring indicates problems that cannot be resolved satisfactorily by remote consultation	9.0 (7.0–9.0)	Consensus reached
**In times of limited healthcare access, what is useful for consultation and communication? Please rate the following**		
Telephone	9.0 (6.5–9.0)	Consensus reached
Video conferencing	7.0 (5.0–9.0)	Consensus reached
Email	6.0 (3.0–8.0)	5.0 (3.0–7.0)
Text messaging	7.0 (3.0–8.0)	Consensus reached
Home visit from HFSN	8.0 (5.0–9.0)	Consensus reached
Authority to determine when clinic or face-to-face practice visit is needed	9.0 (6.5–9.0)	Consensus reached
Virtual multidisciplinary team to discuss case	7.0 (5.0–8.0)	Consensus reached
Having a lead HF consultant for triage	7.0 (5.2–9.0)	Consensus reached
**In a post-pandemic world, healthcare is likely to remain changed and not return to previous practices. Please rate the importance of continuing each item below from 1 (not important at all) to 9 (extremely important) in post-pandemic healthcare**		
Increased use of remote consultations by telephone or video link	8.0 (6.0–9.0)	Consensus reached
Virtual meetings of multidisciplinary teams	8.0 (5.0–8.7)	Consensus reached
Specialist led heart failure secure email system to support GPs and local primary care teams	8.0 (4.2–9.0)	Consensus reached
Strategies to improve social connections form part of annual reviews and clinic visits	7.0 (4.2–8.0)	Consensus reached
Ensuring patients are always asked about loneliness and social connections	7.0 (5.0–8.0)	Consensus reached
Access to heart failure specialist nurses regardless of type of heart failure (preserved and reduced ejection fraction)	8.0 (7.0–9.0)	Consensus reached
Supplying free devices such as weighing scales and blood pressure monitors	7.0 (5.2–9.0)	Consensus reached
Having a secure system for patients and providers to share information	8.0 (7.0–9.0)	Consensus reached
**What would help you (or the patient you care for) sustain a healthy lifestyle? Please rate the importance of each item below from 1 (not important at all) to 9 (extremely important)**		
Financial incentives	3.0 (1.0–5.5)	Consensus reached
Text messages	5.0 (2.0–7.0)	4.0 (2.0–6.0)
Peer support via remote access	6.0 (4.0–8.0)	6.0 (4.0–7.0)
Socially distanced activity with peers	6.5 (6.0–7.0)	6.0 (5.0–7.0)
Fitness trackers	6.0 (4.0–7.0)	6.0 (5.0–7.0)
Telephone calls	6.0 (3.0–7.2)	5.0 (2.0–8.0)
Keeping a record that could be shared	6.0 (4.7–8.0)	6.0 (5.0–8.0)

HFSN = heart failure specialist nurse

Having a safe space to assess patients in person when self-monitoring prompted problems that were unresolvable by remote consultation was deemed very important by both patients and providers (median 9.0, IQR 7.0–9.0). Furthermore, in terms of usefulness for consultation and communication in times of limited healthcare access, consensus was reached in round 1 for telephone (median 9.0, IQR 6.5–9.0), home visits from a heart failure specialist nurse(HFSN) (median 8.0, IQR 5.0–9.0) and the existence of authority to determine when face-to-face practice visits are needed (median 9.0, IQR 6.5–9.0). While this was also the case for text messaging (median 7.0, IQR 3.0–8.0), there was disagreement concerning email (median 6.0, IQR 3.0–8.0). When asked to rate the importance of continuing increased use of remote consultations via telephone or video, both patients and providers deemed it important (median: 8.0, IQR 6.0–9.0) at the point of survey administration.

## Discussion

### Summary

In the UK, the shift towards remote healthcare during the COVID-19 pandemic was pronounced,^16^ The constraints of lockdowns and the risk of virus transmission accelerated the adoption of telehealth solutions,^17^ transforming the approach to managing patients with chronic conditions. Delivering healthcare services at a distance might benefit vulnerable populations and complement in-person HF services.^18^ The pandemic affected the management of people with HF,^19^ necessitating a rapid shift from in-person consultations to remote healthcare interactions. While initially a measure linked to pandemic constraints, this transition unveiled a shift in managing chronic conditions. There is evidence favouring models of care which include virtual consultations, while at the same time retaining the option of in-person consultations if needed.^20,21^

Concern remains about the uneven distribution of digital resources and health disparities across different socioeconomic groups. Studies have shown that during the pandemic, access to telemedicine was affected by differences including older age, minority racial or ethnic identity, female sex, language and socioeconomic differences.^22^

Both patients and providers rated the continued use of remote consultations as important, as well as integrating modern technology in HF management and having spaces for in-person assessments when needed. Although there was generally high consensus that many aspects of remote health communication among providers and patients, and telemedicine were useful and should continue, studies show numerous barriers to telemedicine post pandemic. For example, in 2022, video consultation represented only 2.2% of primary care patient consultations in the UK. Barriers to its use included costs to practice for equipment and lack of consistent integration with primary care electronic systems.^23^ The World Health Organization (WHO) conducted a survey in the WHO European region on digital health use in 2022. Within digital health, using remote monitoring to transmit information to healthcare centres was the most frequently introduced or accelerated during the pandemic. However, post-pandemic barriers such as funding, infrastructure, and capacity and human resources limited the sustained implementation of digital health, and a systems approach was advised to ensure appropriate use and sustainability.^24^

### Strengths and limitations

This study provides insights into challenges and opportunities for future HF healthcare practices. One strength is its comprehensive analysis of healthcare adaptations, including the transition to remote consultations and home monitoring. It highlights innovative responses to unprecedented challenges during the pandemic that could inform the sustained integration of effective innovations into routine HF care. Using a modified Delphi process of gathering consensus among HCPs, patients, and carers ensures findings that are well-rounded and grounded in real-world experiences.

The study has several limitations. The sample size, particularly in the second round of the survey, was small, which limits the generalisability of the results. The reason for the low response rate is not known, but the timing was not ideal given the high demands and stress on the healthcare system. Given the limited response rate, a third survey round was not conducted. The reliance on self-reported data may have led to bias, as participants’ perceptions might not fully reflect actual outcomes. Although the study invited patients with HFpEF who had been involved in previous research, the invitation was also sent to a HF patient charity and providers were encouraged to cascade to patients regardless of HF phenotype. However, it is likely that patient respondents were primarily those with HFpEF who typically have less contact with specialist services. Thus, responses may not reflect the experiences of all patients with HF, although it has been suggested that remote patient management of HF is effective regardless of the phenotype.^25,26^ While the positive impact of remote patient management has been demonstrated in patients with HFand diabetes^27^ there could be differences for other comorbid conditions. We also did not collect sociodemographic information such as ethnicity or socioeconomic status, which may limit the representativeness of the results.

### Comparison with other literature

Most surveys addressing telemedicine and digital health technologies have focused on continuing use and facilitators and barriers to sustainability. Some have solicited patients’ and providers’ perspectives of remote monitoring and care, although this survey is unique in asking about a wider variety of strategies and technology used during the pandemic. A large online survey (*n*=2755 patients and 668 providers) rated remote telerehabilitation as effective, although there was a preference among providers for in-person sessions.^28^ In contrast, an Australian survey and qualitative study of 2525 women and 687 midwives found dissenting opinions as to effectiveness. Although telehealth offered flexibility and convenience, both groups thought it led to inadequate assessment, omissions in care and hindered effective communication.^29^ Qualitative studies of digital health in patients with HF have shown that providers value in-person assessment for risk stratification.^6^

While not yet routinely used in remote HF monitoring and management, wearable technologies,^30^ for instance, show promising potential, particularly when leveraging artificial intelligence.^31^ However, activity trackers were not rated highly in this survey by either patients or providers.

The rapid innovation and implementation brought on by the COVID-19 pandemic has provided a unique opportunity to improve HF management strategies.^32,33^ Remote healthcare services post-pandemic need to consider patient preferences, technological proficiency, and different stages of HF. Personalisation of HF care, including personalised mobile health monitoring systems,^34^ has become crucial to ensure adherence^35^, with challenges that still need addressing.^36^ A systematic review of remote health care for HF highlighted clinical care, communication, education, and ease of use as overarching themes related to engagement.^37^

Remote healthcare has been noted to improve accessibility for some patients while exacerbating the healthcare delivery gap,^38^ and integration of remote healthcare requires efforts to reduce the digital divide.^39^

In the context of virtual wards and hospital-at-home schemes, uncertainties remain regarding their benefits and implementation in clinical practice.^40–42^

Integration into routine service delivery will depend on evidence from real-world settings,^12^ improvements in mortality^43^ and quality of life.^44^ Healthcare systems must develop robust models and protocols^45–47^ that help determine at what point remote consultations have to be complemented by physical assessments.

While serious safety incidents in remote primary care are rare, when they do occur, they can be attributed to various clinical, communicative, technical, and logistical causes within a high-risk context of resource constraints and understaffing.^48^ Identifying and mitigating upstream causes of safety incidents is crucial.^49^

Improvements in the training of HCPs are necessary to manage remote consultations and use telehealth data effectively.^50^

### Implications for research and/or practice

While the pandemic posed significant challenges, it led to a potentially beneficial development in digital health management of people with HF. By integrating advanced technologies and redesigning care models to emphasise flexibility and patient accessibility, healthcare systems can be better prepared for future crises. A thoughtful approach that addresses the potential effectiveness as well as the barriers to and possible inequities of remote healthcare for people with HF and other long-term conditions is essential.

## Data

Anonymised survey data are available from the authors upon reasonable request.
